# Multiple Invasions into Freshwater by Pufferfishes (Teleostei: Tetraodontidae): A Mitogenomic Perspective

**DOI:** 10.1371/journal.pone.0017410

**Published:** 2011-02-25

**Authors:** Yusuke Yamanoue, Masaki Miya, Hiroyuki Doi, Kohji Mabuchi, Harumi Sakai, Mutsumi Nishida

**Affiliations:** 1 Atmosphere and Ocean Research Institute, University of Tokyo, Kashiwa, Chiba, Japan; 2 Natural History Museum and Institute, Chiba, Chiba, Japan; 3 Shimonoseki Marine Science Museum ‘Kaikyokan,’ Shimonoseki, Yamaguchi, Japan; 4 Department of Applied Aquabiology, National Fisheries University, Shimonoseki, Yamaguchi, Japan; Ecole Normale Supérieure de Lyon, France

## Abstract

Pufferfishes of the Family Tetraodontidae are the most speciose group in the Order Tetraodontiformes and mainly inhabit coastal waters along continents. Although no members of other tetraodontiform families have fully discarded their marine lives, approximately 30 tetraodontid species spend their entire lives in freshwaters in disjunct tropical regions of South America, Central Africa, and Southeast Asia. To investigate the interrelationships of tetraodontid pufferfishes and thereby elucidate the evolutionary origins of their freshwater habitats, we performed phylogenetic analysis based on whole mitochondrial genome sequences from 50 tetraodontid species and closely related species (including 31 newly determined sequences). The resulting phylogenies reveal that the family is composed of four major lineages and that freshwater species from the different continents are independently nested in two of the four lineages. A monophyletic origin of the use of freshwater habitats was statistically rejected, and ancestral habitat reconstruction on the resulting tree demonstrates that tetraodontids independently entered freshwater habitats in different continents at least three times. Relaxed molecular-clock Bayesian divergence time estimation suggests that the timing of these invasions differs between continents, occurring at 0–10 million years ago (MA) in South America, 17–38 MA in Central Africa, and 48–78 MA in Southeast Asia. These timings are congruent with geological events that could facilitate adaptation to freshwater habitats in each continent.

## Introduction

Aquatic environments supply aquatic organisms with a large variety of habitats, ranging from deep seas, coral reefs, and coastal and estuarine waters to rivers and lakes. Several barriers have blocked aquatic animals from radiating, among which the interface between saline and freshwater habitats is one of the most formidable barriers. Only limited groups have overcome such boundaries [1]. Gradients in ionic concentration and osmotic pressure have prevented freshwater dispersals of marine animals [2], and moving beyond this interface involves confronting difficulties in the maintenance of a stable internal environment. Even small changes in the ionic balance, osmolality, and pH of body fluids can seriously affect survival [3]. Thus, invasions into freshwater require evolutionary innovation, which has failed to occur in 12 phyla (e.g., Echinodermata, Ctenophora, and Brachiopoda) and many major clades within the remaining phyla [4], [5].

Although freshwater occupies a small portion of Earth's surface (0.8%) [6] and makes up a negligible amount of water on Earth (0.01%), freshwater fishes represent 40–45% of all fish species. Of the freshwater fishes, otophysans (e.g., carps, loaches, characins, catfishes, electric eels) have radiated into freshwater habitats since the Permian [7], [8] and are a major freshwater group that accounts for 64% of freshwater fish species [6], [9], [10]. Euteleostei, the sister group of otocephalans (encompassing the otophysans), is dominant among marine vertebrates and has radiated throughout diverse marine habitats [9]. Some euteleost groups have secondarily radiated into freshwaters [11].

**Figure 1:**
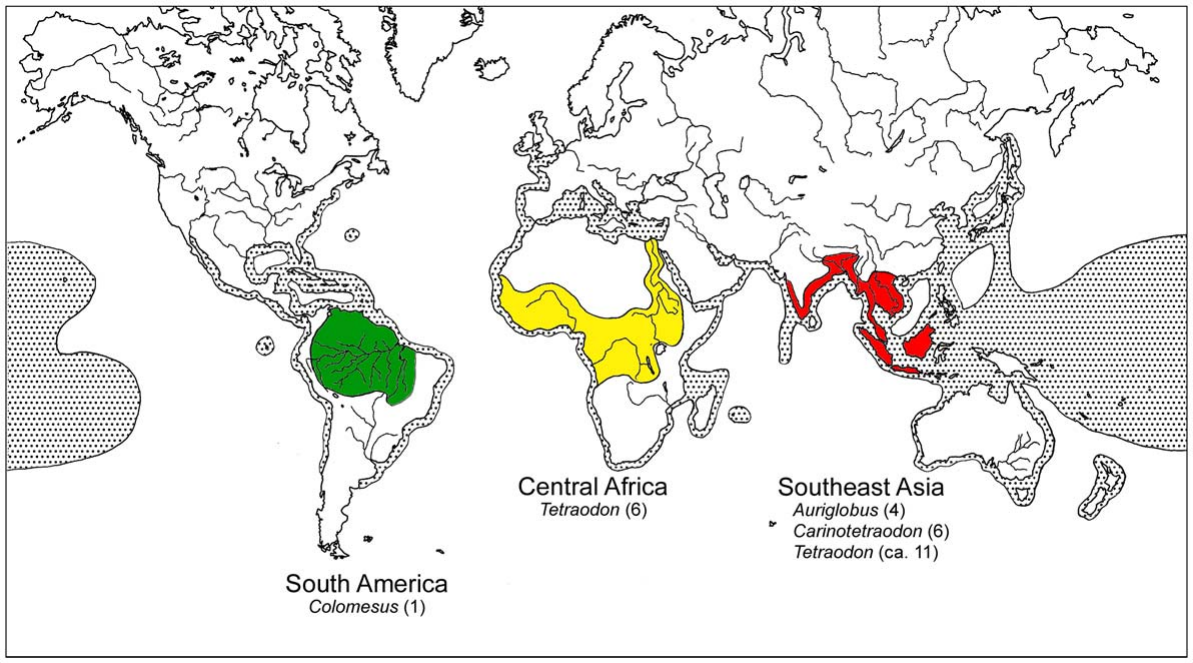
Distribution patterns of marine (shaded) and freshwater (colored) species in Tetraodontidae. Freshwater genera in South America, Central Africa, and Southeast Asia are shown with numbers of species in parentheses. Freshwater regions where only diadromous species occur are not indicated here. Distribution data follow Berra [11], Ebert [18], and Froese and Pauly [12].

The Tetraodontidae, known as pufferfishes, are highly derived euteleosts. Composed of 189 species placed in 19 genera [12], Tetraodontidae is the most speciose family within the Order Tetraodontiformes. Fishes of this family have notably the smallest genomes among vertebrates, approximately 400 Mb or 1/8 the size of the human genome [13]. Considering these features, two pufferfishes, *Takifugu rubripes* and *Tetraodon nigroviridis*, were proposed as model systems for the evolution of vertebrate genomes [14], [15]. Most species occur in inshore and estuarine waters, but approximately 30 tetraodontid species spend their entire life cycles in freshwater in disjunct tropical regions of South America, Central Africa, and Southeast Asia [16], [17], [18] (Figure 1). It should be noted that no fishes in the other tetraodontiform families have discarded their marine lives [9].

To provide an overview of the evolutionary history of freshwater tetraodontids, we used whole mitogenome sequences from 45 tetraodontids (including 31 newly determined sequences) plus those from five outgroups. Unambiguously aligned sequences were subjected to partitioned maximum likelihood (ML) analysis and divergence time estimation. Our results show that tetraodontids entered freshwaters in the different continents during at least three different times and that the timing of these habitat shifts corresponded with geological events in each continent.

## Results

### Genome Organization

Complete L-strand nucleotide sequences from the mitogenomes of the 31 species examined were deposited in the DNA DataBank of Japan (DDBJ), European Molecular Biology Laboratory (EMBL), and GenBank (Table 1). The genome content of the 31 species includes two rRNA, 22 tRNA, and 13 protein-coding genes, plus the putative control region, as is found in other vertebrates. Their gene arrangements are identical to the typical gene order of vertebrates.

**Table 1:** List of species analyzed, with DDBJ/EMBL/GenBank accession numbers. Classification follows Froese and Pauly [12].

Species	Accession No.	Species	Accession No.
Family Triodontidae		*Lagocephalus wheeleri*	AP009538
*Triodon macropterus*	AP009170	*Marilyna darwinii* *	AP011937
Family Molidae		*Omegophora armilla* *	AP011936
*Mola mola*	AP006238	*Polyspina piosae* *	AP011913
*Ranzania laevis*	AP006047	*Pelagocephalus marki* *	AP011938
Family Diodontidae		*Sphoeroides annulatus* *	AP011915
*Diodon holocanthus*	AP009177	*Sphoeroides pachygaster*	AP006745
*Chilomycterus reticulatus*	AP009188	*Sphoeroides parvus* *	AP011914
Family Tetraodontidae		*Sphoeroides testudineus* *	AP011916
*Arothron firmamentum*	AP006742	*Takifugu niphobles*	AP009526
*Arothron hispidus* *	AP011930	*Takifugu oblongus*	AP009535
*Arothron manilensis* *	AP011929	*Takifugu obscurus*	AP009527
*Arothron meleagris* *	AP011931	*Takifugu ocellatus*	AP009536
*Auriglobus modestus* *	AP011917	*Takifugu rubripes*	AP006045
*Canthigaster coronata*	AP006743	*Takifugu xanthopterus*	AP009533
*Canthigaster jactator* *	AP011911	*Tetractenos glaber* *	AP011935
*Canthigaster rivulata*	AP006744	*Tetraodon biocellatus* *	AP011921
*Canthigaster valentini* *	AP011912	*Tetraodon cochinchinensis* *	AP011925
*Carinotetraodon lorteti* *	AP011918	*Tetraodon cutcutia* *	AP011924
*Carinotetraodon salivator* *	AP011919	*Tetraodon nigroviridis*	AP006046
*Chelonodon patoca*	AP009541	*Tetraodon mbu* *	AP011923
*Chelonodon pleurospilus* *	AP011928	*Tetraodon miurus* *	AP011922
*Colomesus asellus* *	AP011909	*Tetraodon palembangensis* *	AP011920
*Colomesus psittacus* *	AP011910	*Torquigener brevipinnis*	AP009537
*Lagocephalus laevigatus* *	AP011934	*Torquigener hypselogenion* *	AP011927
*Lagocephalus lagocephalus* *	AP011933	*Torquigener pleurogramma* *	AP011926
*Lagocephalus sceleratus* *	AP011932	*Tylerius spinosissimus* *	AP011939

*Sequences are newly determined in this study.

### Patterns of Sequence Variations

**Figure 2:**
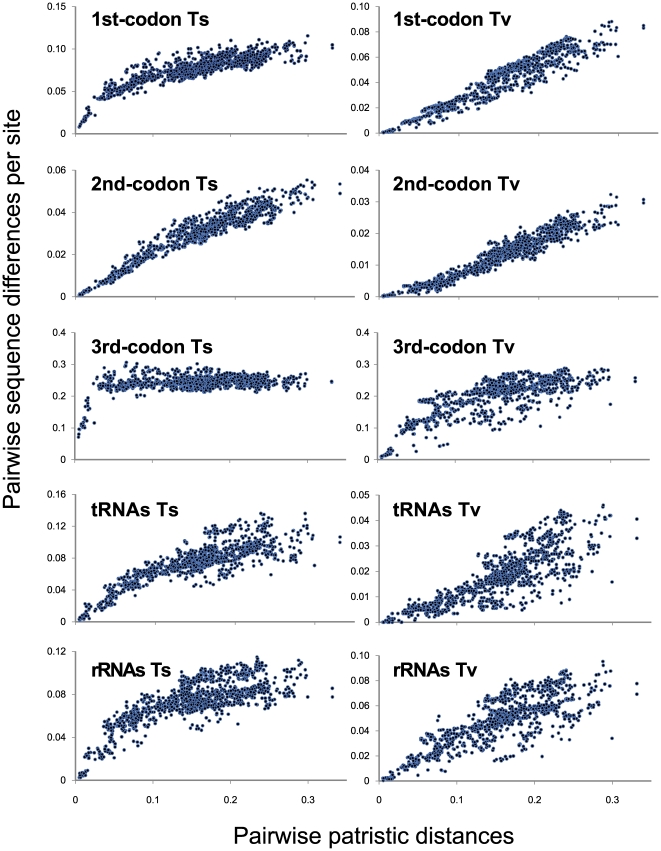
Patterns of sequence variation in the mitochondrial genomes of 45 tetraodontid pufferfishes and five outgroups. Pairwise transitional (TS) and transversional (TV) substitutions per site were plotted against pairwise patristic distances as a substitute for absolute geological time. Gamma-corrected maximum likelihood distances calculated using the mtREV+F model [78] and derived from deduced amino acid sequences for the 13 protein-coding genes were used for evolutionary distances.

Both the pairwise transition (TS) and transversion (TV) differences for each partition increase with increasing evolutionary distance. The exceptions are TS differences at the third codon positions in the protein-coding genes (Figure 2), in which marked saturation is observed in earlier stages of evolution (<0.04 evolutionary distance), with no increase observed thereafter. In addition, TS differences at the first codon positions of the protein-coding genes, tRNA genes, and rRNA genes, and TV differences at the third codon positions of the protein-coding genes exhibit gradual saturation curves in distant pairs, although pairwise differences seem to steadily accumulate along the time axis (Figure 2).

### Phylogenetic Relationships

**Figure 3:**
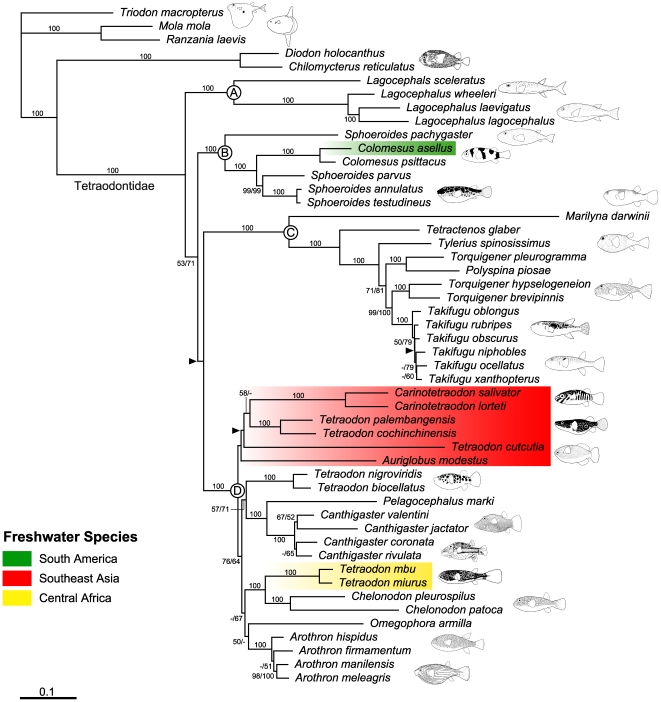
An ML tree with estimated branch lengths using the 12_n_3_r_RT_n_ dataset. Topological differences between the ML analysis for the 12_n_3_r_RT_n_ and 123_n_RT_n_ datasets are indicated by arrowheads. Numbers near internal branches indicate ML bootstrap probabilities for the 12_n_3_r_RT_n_ (left) and 123_n_RT_n_ (right) datasets (values less than 50% not shown). Single numbers indicate that these estimates were identical.

Based on the patterns of sequence variation, the dataset with the third codon positions converted by RY-coding (12_n_3_r_RT_n_) was expected to effectively remove the noise expected from quickly saturated transitional changes in the third codon positions and avoid a lack of signal by retaining all available positions in the dataset [19], [20]. Accordingly, partitioned ML analysis based on the 12_n_3_r_RT_n_ dataset is shown in Figure 3.

Our analysis confidently recovered monophyly of Tetraodontidae as well as its sister-group relationship with Diodontidae with 100% bootstrap probabilities (BP) (Figure 3). Tetraodontidae is composed of four major lineages (Clades A–D) that are supported by 100% BPs. Although basal relationships among these four clades are ambiguous, Clade A is the most basal, followed by the divergences of Clade B and Clades C+D.

Regarding the freshwater species, a South American species (*Colomesus asellus*) is deeply nested in Clade B together with a congeneric, euryhaline species (*C. psittacus*). Central African and Southeast Asian freshwater species fall into two different clades that are nested within Clade D (Figure 3), although both groups include species of *Tetraodon*. Two species of Central African *Tetraodon* form a well-supported clade (BP = 100%), with a sister-group relationship with *Chelonodon* species (BP = 100%) (Figure 3). Southeast Asian freshwater species also form a clade in the resulting phylogenies, but this clade is weakly supported (BP<50%) (Figure 3).

Three genera, *Tetraodon*, *Torquigener*, and *Sphoeroides*, were found as non-monophyletic, and monophyly of each genus was confidently rejected by the approximately unbiased (AU) test (*P* = 0.000).

### Divergence Times of Tetraodontid Pufferfishes

**Figure 4:**
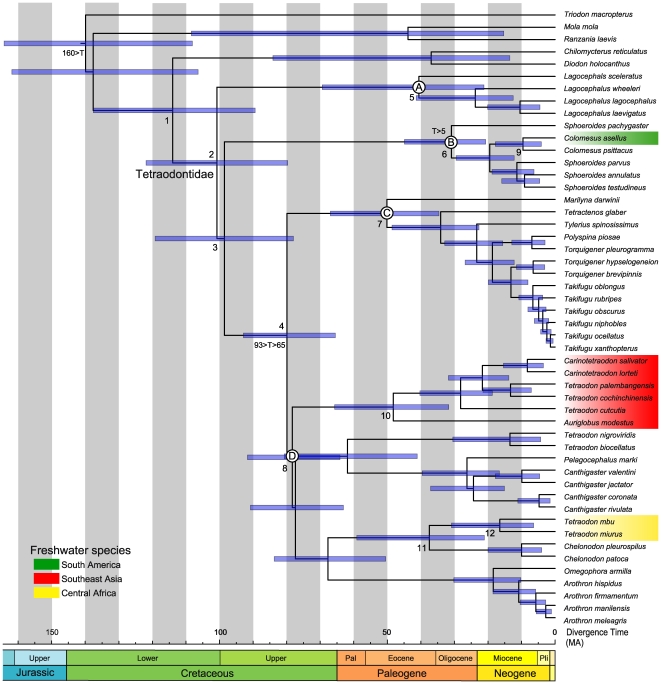
Divergence times among tetraodontid pufferfishes from the relaxed-molecular clock method with the dataset 12_n_RT_n_. Node numbers 1–12 and three time constraints are indicated above and below nodes, respectively. Horizontal bars indicate 95% credible intervals of the divergence time estimates.

MCMCTREE analysis of divergence times is shown in Figure 4 and Table 2 (Figure S1 and Table S1 for more information), with the 95% credible interval (CI). Tetraodontids are estimated to diverge from diodontids at 114 million years ago (MA) (89–138 MA), and the four major lineages of tetraodontid pufferfishes (Clades A–D) diverged from the ancestral lineage during the late Cretaceous (80–101 MA). The most recent common ancestors of Clades A–D date back to 41 MA (21–69 MA), 31 MA (21–45 MA), 50 MA (35–67 MA), and 78 MA (65–93 MA), respectively (Figure 4, Table 2). The South American freshwater species, *Colomesus asellus*, diverged from *C. psittacus* at 10 MA (4–18 MA) in Clade B (Figure 4, Table 2). Southeast Asian freshwater species diverged from the remaining members of Clade D at 78 MA (65–93 MA), and their most recent common ancestor emerged at 48 MA (32–66 MA) (Figure 4, Table 2). Central African freshwater species diverged from *Chelondon* species at 38 MA (21–59 MA), and their most recent common ancestor emerged at 17 MA (6–31 MA) (Figure 4, Table 2).

**Table 2:** Posterior means and 95% credible intervals of divergence time estimates (MA) of 12 nodes of the tree shown in Figure 4, based on the relaxed molecular-clock analysis for the 12_n_RT_n_ dataset.

Node	Posterior mean	95% credible interval
1	114.0	89.4–137.7
2	100.8	79.7–121.9
3	98.6	78.0–119.2
4	79.9	65.5–92.9
5	40.6	21.2–69.4
6	30.9	20.7–44.9
7	50.2	34.6–67.0
8	78.4	64.1–91.7
9	9.6	4.1–17.8
10	48.3	31.7–65.7
11	37.5	1.1–59.1
12	16.5	6.4–30.9

### Habitat Evolution

**Figure 5:**
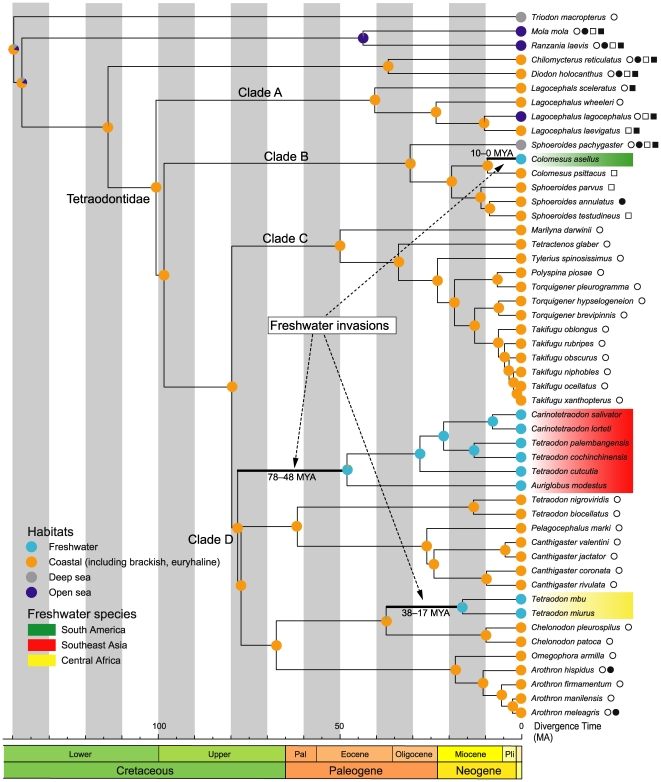
ML reconstruction of habitat types mapped onto the ML tree from the 12_n_3_r_RT_n_ dataset. Support for each character state is indicated in each node. Branches proposed for the occurrence of freshwater invasions are bolded and numbers besides the branches indicate estimated intervals for the invasion. Open and solid circles and open and solid squares indicate species distributions in the Indo-West Pacific (including Polynesian Pacific), East Pacific, West Atlantic, and East Atlantic (including Mediterranean), respectively.

The ML reconstruction for ancestral habitats (coastal = character state 0; freshwater = state 1; deep sea = state 2; and open sea = state 3) in tetraodontid pufferfishes is shown in Figure 5. The ancestral habitat of the most recent common ancestor for tetraodontid pufferfishes is most likely coastal (*P*_0_ = 0.998); this habitat is found in all four major lineages in the family and is also dominant among both extant members and ancestral nodes (Figure 5). The open sea and deep sea habitat states only occur locally in Clades A and B, respectively, and these two habitats are not primary character states for each ancestral node (Figure 5). The freshwater habitat is the second most common character state and is a primary character state for extant species and common ancestral nodes within Central African (*P*_1_ = 0.980) and Southeast Asian (*P*_1_ = 0.945) freshwater clades (Figure 5). According to these results, tetraodontid pufferfishes shifted their habitats from coastal waters into open seas, deep seas, and freshwaters, not vice versa, and they also independently invaded freshwaters from coastal habitats in different continents at least three separate times (Figure 5).

## Discussion

### Phylogeny of Tetraodontid Pufferfishes

Tetraodontid pufferfishes have long attracted biologists across diverse fields, such as morphology, toxicology, and molecular biology, but their phylogenetic relationships have remained unexplored. A morphology-based classification that divides this family into two subfamilies, Canthigasterinae (containing only species of the genus *Canthigaster*) and Tetraodontinae [21], [22], [23], is confidently rejected by the AU-test (*P* = 0.000), as also reported in previous molecular studies [24], [25], [26]. In addition to such a subfamilial classification, the following phylogenetic hypotheses for this group were proposed based on either morphology or molecules. Tyler [23] provided osteological and external morphological descriptions of representative species of tetraodontid genera and accordingly illustrated a tetraodontid phylogeny of 16 genera, although without the use of cladistic methods. Holcroft [24] and Alfaro et al. [25] attempted to resolve the phylogeny of the entire Tetraodontiformes by using nuclear RAG1 and mitochondrial 12S and 16S rRNA gene sequences, and their datasets included 19 tetraodontid species, respectively. Based on morphological similarities, Tyler [23] recognized the four major clades of tetraodontids that were found in the present study, and Holcroft [24] and Alfaro et al. [25] also produced phylogenetic structures that are largely congruent with our resulting topology, in that all four lineages are reproduced. It should be noted that the four main lineages of tetraodontid pufferfishes show distinctive geographic patterns. Clades A, C, and D have centers of distribution in the tropical and temperate Indo-West Pacific, while Clade B has a center of distribution in the tropical and temperate West Atlantic and East Pacific (Figure 5).

Three genera, *Tetraodon*, *Torquigener*, and *Sphoeroides*, were found as non-monophyletic, and monophyly of each genus was confidently rejected by the approximately unbiased (AU) test (*P* = 0.000). Phylogenies of these genera have never been investigated by either morphology or molecules and generic classification should be revised in future studies.

### Divergence times of Tetraodontid Pufferfishes

Our results for the timing of divergences are largely congruent with previous mitogenomic studies [27], [28], but they are much older than estimates from fossil records [29], [30], [31] and timescales based on the nuclear RAG1 gene [32], [33]. This discrepancy regarding the teleostean timescale is regarded as an effect of higher rates of evolution in mitogenomes from the viewpoint of fossils and nuclear genes [34]. However, using the RAG1 gene, Santini et al. [32] and Alfaro et al. [33] estimated ages for important percomorph clades, the tetraodontiforms and cichlids, which are inconsistent with evidence from biogeography and fossil records. The divergence time of tetraodontiform fishes from their sister lineage was estimated at about 75 MA despite the fact that the oldest fossils were present at 95 MA [35]. The advent of cichlids was estimated at 59 MA (46–73 MA), which was long after the Gondwanian breakup, at around 100 MA [36], despite their Gondwanian distribution pattern [7], [37] and the lack of evidence for intercontinental dispersal [38], [39].

Mitogenomic hypotheses on teleostean timescales also show strong discrepancies from fossil evidence [27], [37]. However, the earliest fossil records do not necessarily indicate that a group diverged from its sister group at that time. Particularly, fossils of teleosts are incomplete in various respects and the long-term lack of teleostean fossils can be explained by the influence of population size and taphonomic conditions for fossilization [40].

### Freshwater Invasions

The distribution patterns of primary freshwater fishes have provided good evidence for the geological reshaping of land and freshwater systems because primary freshwater fishes have low tolerance to salt water and cannot disperse beyond seaways (e.g., [8], [10], [41], [42]). Conversely, secondary freshwater fishes can survive in salt water, to an extent, and interpretations of their distribution patterns in terms of vicariance biogeography have often been controversial, even when using molecular-based methods (e.g., [37], [43], [44], [45]). Although South America, Africa, and India, where freshwater pufferfishes occur, were components of Gondwana, the phylogenetic structure, divergence times, and reconstructed ancestral habitats obtained in the present analysis indicate that the common ancestor of freshwater pufferfishes invaded freshwater independently in these continents. Monophylies of all of the freshwater species, and all pairwise combinations of freshwater species on these continents (South America and Central Africa, South America and Southeast Asia, Central Africa and Southeast Asia), were confidently rejected by the AU-test (*P* = 0.000). The ML reconstruction of ancestral habitats demonstrates that the tetraodontids independently invaded freshwater habitats in the different continents at least three separate times (Figure 5). The timings of such invasions were different for each continent, around 0–10 MA in South America, 17–38 MA in Central Africa, and 48–78 MA in Southeast Asia (Figures 4 and 5, Table 2).

Most coastal pufferfishes are reported to temporarily occur in brackish and freshwater regions for rest or refuge [18], [46], and many pufferfishes spend some of their life stages in freshwater environments. For example, two *Takifugu* species, *T. obscurus* and *T. ocellatus*, have an euryhaline nature and migrate up rivers for spawning [3], [47], [48]. Also, some species of *Chelonodon* and *Arothron* spend their juvenile stages in the mouth or even in the middle of rivers and estuaries [46], [49]. Furthermore, non-freshwater species of *Tetraodon* (e.g., *T. nigroviridis* and *T. biocellatus*), which are often mistakenly regarded as freshwater pufferfishes, have considerable resistance to freshwater exposure and are widely sold in the freshwater aquarium trade [18], [46]. All four clades include marine species that have been found in brackish and/or freshwater regions [12], [18], and many marine species have some resistance to freshwater exposure [46]. Compared to our results, the resistance of marine puffers to freshwater exposure does not strongly correlate with their phylogenetic position. Nevertheless, our results indicate that full-scale freshwater invasions of pufferfishes occurred rarely, not repetitively, suggesting the difficulties encountered by marine pufferfishes when invading occupied niches in freshwater environments. Thus, it seems likely that invasions into freshwater by marine species might be explained by unusual events. The timings of freshwater invasions were congruent with geological events in the respective regions, which could facilitate adaptation to freshwaters.

### South America

*Colomesus asellus* is widely distributed in the Amazon River basin from Peru to Marajó Island, including tributaries of the Araguaia and Guaporé rivers, the lower reaches of the Orinoco River basin, and the Essequibo River basin [12], [50]. The sister species *C. psittacus* occurs in brackish and coastal waters around the north of South America [16], [50] and is somewhat resistant to freshwater [18]. These two species have strong morphological similarities [16], but our results show that they diverged from each other at 10 MA (Figure 5). During the Miocene (ca. 9–17 MA), a lacustrine or wetland system that drained northward to the Caribbean Sea had developed in the western Amazonian, and marine incursions occurred twice during this period [51], [52]. A subsequent rise of the northern Andes and incidental regions transformed this lacustrine basin into the Amazon and Orinoco river systems, with the present directions of flow, at around 8–10 MA, in the late Miocene [53], [54], [55], [56]. Miocene marine incursions in this region are known to have facilitated the evolutionary transition of various groups from marine to freshwater habitats [51], [57], [58], [59], [60]. The present results for the divergence time between *Colomesus* species indicate that their ancestors invaded the western Amazonian through the latter Miocene marine incursion (11–12 MA) and subsequently diverged from the marine and brackish lineage with adaptation to freshwater habitats.

### Central Africa

In Central Africa, six species of *Tetraodon* exclusively inhabit freshwater environments. *Tetraodon duboisi*, *T. mbu*, *T. miurus*, and *T. schoutedeni* are distributed in the Congo basin, *T. pastulatus* is found in the Cross River in the southeastern Nigerian delta, and *T. lineatus* has a much wider occurrence, ranging from the Senegal and Volta basins to the Niger and Nile basins and Lake Turcana [18]. Major marine incursions into Africa are thought to have occurred repeatedly in the Mesozoic and Cenozoic [61], [62], [63], and increases in sea level due to climate fluctuations repeatedly spurred marine incursions after the Jurassic. A large inland sea extended from Libya to West Africa from the late Cretaceous to the Eocene, according to sedimentological analyses of Central and West Africa [62]. Our results indicate that African freshwater invasions of *Tetraodon* species occurred between 17–38 MA (Figure 5), which is consistent with the end of the major marine incursions in this region during the early and middle Eocene (37–54.8 MA) [62]. Therefore, it seems likely that *Tetraodon* invaded freshwater not from the Atlantic coasts but from the northern coast of Africa, which then abutted the Tethys Sea. This is congruent with the fact that the rivers that these species inhabit do not flow into the Indian Ocean, despite the fact that their sister group, *Chelondon*, only occurs in the Indo-West Pacific. It has been hypothesized that freshwater herrings also invaded African freshwaters from marine environments because of this marine incursion [64].

### Southeast Asia

Southeast Asian freshwater pufferfishes include approximately 21 species in three to five genera, which are distributed from Southwest India to Indochina and the Sunda Islands. They have speciated considerably in Indochina and the Sunda Islands, particularly in the Mekong Basin, while *Carinotetraodon imitator*, *C. travancoricus*, and *Tetraodon cutcutia* are found in the Indian subcontinent. Our analysis indicates that Southeast Asian freshwater pufferfishes are represented by four lineages: *Carinotetraodon* spp. (South India to Sunda Islands), *Auriglobus* spp. (Indochina, Malay peninsula, and Sunda Islands), *Tetraodon cutcutia* (East India to Myanmar), and other *Tetraodon* species (Indochina, Malay Peninsula, and Sunda Islands) [12], [17], [18]. Our results also indicate that the freshwater invasion by their common ancestor occurred sometime between the late Cretaceous and the early Eocene (48–78 MA) (Figure 5). However, because their monophyly is supported by very weak probabilities (BP<50%), we cannot rule out the possibility of their non-monophyly and the occurrence of multiple invasions into the freshwater habitats of Southeast Asia. If the hypothesis of a single freshwater invasion is true, the timing of the invasion would have corresponded with the period when the Indian subcontinent and Asia approached very close to and had just collided with each other. The Indian subcontinent drifted northward and collided with the southern coast of Tibet around the early or middle Eocene (ca. 50 MA) [65], [66]; the northward thrust was due to the drift of the Indian Plate and caused the uplift of the Tibetan Plateau [67], [68]. In the Cretaceous, southern Tibet was covered by shallow marine water [69], and as Tibet was uplifted, marine environments in eastern central Tibet shifted into narrow lacustrine basins in the Paleocene and Eocene [70]. Therefore, we can hypothesize that the common ancestors of Southeast Asian freshwater pufferfishes inhabited these shallow marine regions in Tibet; during the Paleocene and Eocene, they became trapped in lacustrine basins that formed from the shallow marine waters and subsequently became adapted to freshwater habitats. At present, the upper reaches of the Brahmaputra, Irrawaddy, Salween, Mekong, and Yangtze rivers flow through eastern Tibet, and freshwater pufferfishes occur in most of these rivers. Freshwater pufferfishes do not occur in the Yangtze River, but the upper reaches of the Yangtze River system flowed into the South China Sea through the current Red River Basin in northeastern Indochina before Oligocene-Miocene time (ca. 10–30 MA) [71]. The occurrence of these fishes in the Sunda Islands probably resulted from lowering sea levels during the Pleistocene glacial era, that reached levels 120 m below present, and fishes could travel into the Sunda Islands without moving beyond the sea [72]. Because there is weak support for their monophyly, this hypothesis regarding their freshwater invasion and radiation should be confirmed in the future by more extensive taxonomic sampling.

### Conclusion

Phylogenetic analysis of whole mitogenomic datasets reveals that tetraodontid species that spend all of their life stages in freshwater in South America, Central Africa, and Southeast Asia are independently nested in two of the four lineages, and the ML reconstruction of ancestral habitat states demonstrates that tetraodontids independently invaded freshwater habitats in the different continents at least three separate times. The relaxed molecular clock analysis of divergence times showed that the timings of these invasions were different for each continent, occurring around 0–10 MA in South America, 17–38 MA in Central Africa, and 48–78 MA in Southeast Asia. These timings are congruent with geological events, such as marine incursions, that could facilitate adaptation to freshwaters. Although many pufferfishes have some resistance to freshwater exposure, invasions into freshwater habitats occurred rarely, not repetitively. This suggests the difficulties faced by marine pufferfishes when invading occupied niches in freshwater environments.

Of the currently recognized 28 freshwater species (South America: 1 sp.; Central Africa: 6 spp.; Southeast Asia: 21 spp.; see Figure 1), we used only nine freshwater species (South America: 1 sp.; Central Africa: 2 spp.; Southeast Asia: 6 spp.). Although our analysis presents a new perspective on the phylogeny and evolution of tetraodontid pufferfishes, we cannot rule out the possibility that this incomplete taxonomic sampling causes estimation errors on phylogeny and divergence times of freshwater species. Accordingly, more extensive taxonomic sampling of these groups is necessary to more precisely reconstruct their evolutionary history and revise their taxonomy and classification.

## Materials and Methods

### Ethics Statements

This project was conducted in accordance with the Regulations for Animal Experiments of the University of Tokyo. An ethics statement is not required for this project.

### Taxonomic Sampling

We tried to choose at least two species for each genera, except for monotypic genera; however, only one species was available for several genera, including *Auriglobus*, *Marilyna*, *Omegophora*, *Pelagocephalus*, and *Tetractenos*. Three to seven species were used for speciose genera such as *Arothron*, *Canthigaster*, *Lagocephalus*, *Sphoeroides*, *Takifugu*, *Tetraodon*, and *Torquigener*. Final rooting was done using *Triodon macropterus* (Triodontidae), *Mola mola* and *Ranzania laevis* (Molidae), and *Diodon holocanthus* and *Chilomycterus reticulatus* (Diodontidae), based on the results of Yamanoue et al. [26]. Table 1 lists all species used in this study, with their DDBJ/EMBL/GenBank accession numbers.

### DNA Extraction, PCR, and Sequencing

A portion of the epaxial musculature or fin clips (∼0.25 g) was excised from fresh specimens of each species and immediately preserved in 99.5% ethanol. Total genomic DNA was extracted using a Qiagen DNeasy tissue kit (Qiagen) following the manufacturer's protocol. The mitogenomes were amplified in their entirety using a long PCR technique [73]. A total of 12 fish-versatile long PCR primers were used in various combinations to amplify the entire mitogenome. The long PCR products were diluted with TE buffer (1∶19) for subsequent analyses as PCR templates.

A total of 181 fish-versatile PCR primers were used in various combinations to amplify the contiguous, overlapping segments of the entire mitogenome, and 20 species-specific primers were designed when no appropriate primers were available. A list of the PCR primers used in this study is available from Y.Y. upon request.

Long PCR and subsequent short PCR were performed as previously described [74], [75]. Double-stranded PCR products, purified using an ExoSAP-IT (USB), were subsequently used for direct cycle sequencing with dye-labeled terminators (Applied Biosystems). The primers used were the same as for PCR. All sequencing reactions were performed according to the manufacturer's instructions. Labeled fragments were analyzed using Model 3130 DNA sequencers (Applied Biosystems).

### Alignment

The DNA sequences were edited and analyzed with EditView v.1.0.1, AutoAssembler v.2.1 (Applied Biosystems) and DNASIS v.3.2 (Hitachi Software Engineering). A total of 13 protein-coding, 22 tRNA, and two rRNA gene sequences for 31 species were aligned using ProAlign v.0.5 [76]. All sequences from L-strand-encoded genes (ND6 and eight tRNA genes) were converted to complementary strand sequences. Amino acids were used for alignments of protein-coding genes. Regions with posterior probabilities of >70% were used in the phylogenetic analyses. Unambiguously aligned sequences were identified at 11,328, 1454, and 2613 nucleotide positions for the 13 protein-coding genes, 22 tRNA genes, and two rRNA genes, respectively (total of 15,395 positions).

We constructed two different datasets to investigate the effects of quickly saturating the third codon positions in the protein-coding genes on the phylogeny estimation: 1) all aligned positions of gene-coding regions of mitogenomic sequences (designated as 123_n_RT_n_, where n denotes nucleotides) and 2) the third codon positions converted to purine (R) and pyrimidine (Y) (12_n_3_r_RT_n_, where r denotes RY-coding [19], [20]). In the divergence time analysis, the third codon positions were excluded from the dataset (12_n_RT_n_: 11,619 positions) to remove the effects of saturation on branch length estimation. The aligned sequence data in NEXUS format are available from Y.Y. upon request.

### Analysis of Sequence Variations

Pairwise comparisons and statistical information from the mitogenomic sequences were obtained using PAUP v.4.0b10 [77]. To examine patterns of sequence variation in the first, second, and third codon positions, and separately for the protein-coding genes, rRNA, and tRNA, we plotted pairwise nucleotide differences (sorted into transitional [TS] and transversional [TV] differences) against evolutionary distance as a substitute for absolute geological time. The gamma-corrected ML distance with the mtREV + F model [78] derived from concatenated amino acid sequences from the 13 protein-coding genes was calculated with TREE-PUZZLE v.5.2 [79] and used as the evolutionary distance. Distances resulting from this method have been demonstrated to be linear with absolute geological time for several vertebrate taxa [80].

### Phylogenetic Analysis

The aligned datasets were subjected to an ML analysis. We set five partitions for the unambiguously aligned nucleotide sequences of the 123_n_RT_n_ and 12_n_3_r_RT_n_ datasets, assuming that functional constraints on sequence evolution are more similar within codon positions (or types of molecule) across genes than across codon positions (or types of molecule) within genes, at least for a set of mitochondrial genes. ModelTest v.3.0.6 [81] selected GTR+I+Γ [82] as the optimum model for sequence evolution based on Akaike's information criterion (AIC) for all partitions from the 123_n_RT_n_ dataset. The ML analysis was conducted with RAxML v.7.2.6 [83], setting GTRGAMMAI as a nucleotide substitution model for the GTR+I+Γ model. We reconstructed an ML tree, and a rapid bootstrap analysis with 1000 replications (–f a option) was simultaneously performed to calculate the robustness of each branch of the resultant tree.

### Testing Alternative Hypotheses

Alternative tree topologies were individually compared to the resulting ML tree from the 12_n_3_r_RT_n_ dataset using the likelihood-based approximately unbiased (AU) test [84] implemented in CONSEL v.0.1k [85]. ML analyses using RAxML with constrained topology were conducted and ML scores were estimated using the GTR+I+Γ model of sequence evolution. Differences between constrained topologies and the resultant tree were statistically evaluated. A value of *P*<0.05 was considered significantly different.

### Divergence Time Estimation

To investigate the relative timings of major cladogenetic events in the family Tetraodontidae, a relaxed molecular-clock Bayesian method implemented in MCMCTREE in PAML v.4.4 [86] was used for dating analysis. The best-scoring ML tree from the 12_n_3_r_RT_n_ dataset was used for divergence time estimation, and ML estimates of branch lengths were obtained using the BASEML program in PAML under the GTR+Γ substitution model with the gamma prior set at 0.2. Two priors, the overall substitution rate (rgene gamma) and the rate-drift parameter (sigma2 gamma), were set at *G* (1, 7.9) and *G* (1, 1.0), respectively, using the strict molecular-clock assumption with a 75 MA constraint (a result of Azuma et al. [37]) on the divergence between *Takifugu rubripes* and *Tetraodon nigroviridis*. The independent-rates (IR) model [87] was used to specify the priors of rates among internal nodes (clock = 2 in MCMCTREE). Recent research [88] has suggested that the IR model is more appropriate than the correlated-rates model for divergence time estimation [88]. The parameters of the birth-death process for tree generation with species sampling [89] were fixed at *λ* = *μ* = 1 and *ρ* = 0, so that the priors were similar to those used in previous mitogenomic studies using MULTIDIVTIME [27], [37], [90]. A loose maximum bound for the root was set at <2.0 ( = 200 MA).

The MCMCTREE program allows for minimum (lower) and maximum (upper) time constraints, and it has been argued that multiple calibration points provide more realistic divergence time estimates overall [91]. Therefore, we sought to obtain an optimal phylogenetic coverage of calibration points across our tree, although few good calibration points were found based on fossils or biogeography within Tetraodontidae. In addition, previous studies provided disparate divergence time estimates. Yamanoue et al. [27] estimated the divergence between *Tetraodon nigroviridis* and *Takifugu rubripes*, and between Tetraodontiformes and its sister group Caproidei, at 73 MA (with a 95% CI of 57–94 MA) and 160 MA (with a 95% CI of 133–194 MA), respectively, using whole mitogenome sequences and multiple fossil calibration points with a partitioned Bayesian method [92]. The former estimate was later confirmed by Azuma et al. [37] (75 MA with a 95% CI of 65–93 MA), who used the same method with more taxa and more calibration points. Thus, 160 MA and 65–73 MA, from the results of Yamanoue et al. [27] and Azuma et al. [37], were used as the time constraints for the root age and the divergence between *Takifugu* and *Tetraodon* (Figure 4). Moreover, despite the poor fossil records of tetraodontids, a fossil record of *Sphoeroides* from the Pliocene (5 MA) [93] was used as a calibration for the root node of Clade B (Figure 4)

A softbound version of the program (MCMCTREE) was used, so that probabilities of the true divergence time that fell outside the maximum and minimum bounds were not zero [94]. All time constraints are provided in a unit of 100 MA (i.e., 1 = 100 MA) because some of the model components in the Bayesian analysis are scale variant and the node ages should fall between 0.01 and 10 [86]. A time constraint setting assumes a heavy-tailed density based on a truncated Cauchy distribution of *p* = 0.1 and *c* = 1 as the default [86]. MCMC approximation with a burn-in period of 150,000 cycles was obtained, and every 50 cycles were taken to create a total of 30,000 samples. To diagnose possible failures of the Markov chains to converge to their stationary distribution, at least two replicate MCMC runs were performed with two different random seeds for each analysis. In addition, distributions of parameter values from MCMC samples were visualized using Tracer v.1.5 (available from http://tree.bio.ed.ac.uk/software/tracer/) to check mixing, choose a suitable burn-in, and look for trends that might suggest problems with convergence. The number of samples (30,000) was large enough to reach effective sample sizes (ESSs>200) for all parameters estimated in this study.

### Estimation of Habitat Evolution

Habitats of tetraodontid pufferfishes were variable, ranging from freshwater, brackish and estuarine water, and coastal water, to the bottom of the continental shelf and open sea. In this analysis, their habitats were simply classified into four categories for convenience. First, species that spend all of their life stages in freshwater were categorized as having a freshwater habitat. The ecology of tetraodontid pufferfishes that inhabit coastal waters varies greatly with species. Some spend part of their life in brackish and freshwater regions, while others rarely enter freshwater [18]. Current knowledge about their life history is not sufficient to sort these species into more subdivided categories. Therefore, they were classified as having a coastal water habitat. A few species spend most of their lives off shore, and their habitats can be divided into the bottom of the deep sea and pelagic waters of the open sea. All species used in this study were classified into freshwater, coastal water, deep sea, and open sea habitats, which were determined according to Ebert [18] and Froese and Pauly [12]. To examine the evolution of habitat use in tetraodontid pufferfishes, ancestral states were estimated by calculating likelihood scores using Mesquite v.2.73 [95] with the MK1 model. This estimation was based on the ML tree from the 12_n_3_r_RT_n_ dataset with branch lengths fixed by MCMCTREE, and the likelihood scores of these four character states were computed for each node of the tree.

## Supporting Information

Figure S1 Node numbers on the best-scoring ML tree for showing divergence time estimates in Table S1.
(TIF)

Table S1 Summary of MCMC samples for node ages (MA) in the independent-rates analyses using MCMCTREE. For node numbers, see Figure S1.
(DOC)
