# Supplementary figures and images for: Multiple Invasions into Freshwater by Pufferfishes (Teleostei: Tetraodontidae): A Mitogenomic Perspective
Source: PLoS One. 2011 Feb 25;6(2):e17410. doi: 10.1371/journal.pone.0017410 (PMC3045446; doi:10.1371/journal.pone.0017410)

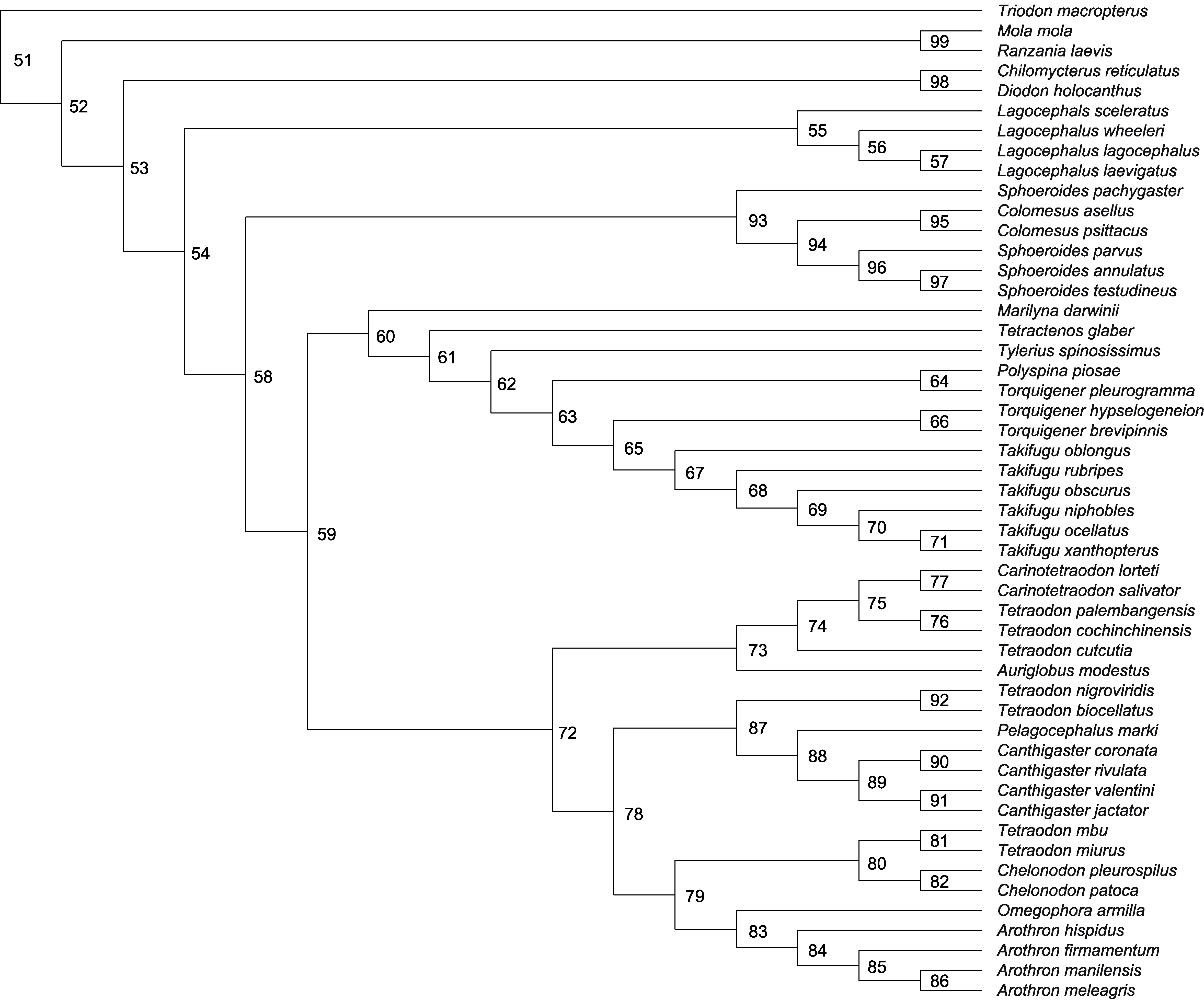

Supplement: Figure S1 — Node numbers on the best-scoring ML tree for showing divergence time estimates in Table S1. (TIF) [file pone.0017410.s001.tif]
