# Supplementary material for: Multiple Invasions into Freshwater by Pufferfishes (Teleostei: Tetraodontidae): A Mitogenomic Perspective
Source: PLoS One. 2011 Feb 25;6(2):e17410. doi: 10.1371/journal.pone.0017410 (PMC3045446; doi:10.1371/journal.pone.0017410)
Supplement: Table S1 — Summary of MCMC samples for node ages (MA) in the independent-rates analyses using MCMCTREE. For node numbers, see Figure S1. (DOC) [file pone.0017410.s002.doc]

**Table S1.**  Summary of MCMC samples for node ages (MA) in the independent-rates analyses using MCMCTREE. For node numbers, see figure S1.

| Node | Mean | 95% | 95% | Node | Mean | 95% | 95% |
| --- | --- | --- | --- | --- | --- | --- | --- |
|  |  | (lower) | (upper) |  |  | (lower) | (upper) |
| 51 | 141.48 | 108.14 | 164.19 | 76 | 13.29 | 7.11 | 21.51 |
| 52 | 137.71 | 106.40 | 161.90 | 77 | 8.27 | 3.48 | 15.40 |
| 53 | 114.03 | 89.44 | 137.65 | 78 | 77.44 | 63.13 | 90.82 |
| 54 | 100.83 | 79.68 | 121.88 | 79 | 67.73 | 50.46 | 83.72 |
| 55 | 40.64 | 21.24 | 69.41 | 80 | 37.52 | 21.08 | 59.10 |
| 56 | 23.77 | 12.51 | 41.42 | 81 | 16.48 | 6.43 | 30.92 |
| 57 | 10.48 | 4.52 | 20.11 | 82 | 10.02 | 4.04 | 19.96 |
| 58 | 98.61 | 78.04 | 119.16 | 83 | 18.45 | 10.15 | 30.21 |
| 59 | 79.94 | 65.47 | 92.89 | 84 | 10.84 | 5.75 | 18.53 |
| 60 | 50.17 | 34.60 | 67.04 | 85 | 5.76 | 2.82 | 10.35 |
| 61 | 34.12 | 22.73 | 48.57 | 86 | 2.78 | 1.11 | 5.57 |
| 62 | 23.36 | 15.59 | 32.89 | 87 | 61.94 | 41.11 | 80.72 |
| 63 | 18.68 | 12.19 | 26.76 | 88 | 26.30 | 16.63 | 39.58 |
| 64 | 6.93 | 2.96 | 12.93 | 89 | 24.34 | 15.09 | 37.06 |
| 65 | 13.16 | 8.10 | 19.88 | 90 | 4.76 | 1.52 | 11.09 |
| 66 | 6.53 | 3.09 | 11.51 | 91 | 9.92 | 4.64 | 17.79 |
| 67 | 6.62 | 3.70 | 10.79 | 92 | 13.49 | 4.32 | 30.46 |
| 68 | 4.91 | 2.67 | 8.07 | 93 | 30.93 | 20.67 | 44.94 |
| 69 | 3.69 | 1.93 | 6.19 | 94 | 19.49 | 12.19 | 29.54 |
| 70 | 2.44 | 1.19 | 4.30 | 95 | 9.60 | 4.06 | 17.76 |
| 71 | 1.41 | 0.59 | 2.70 | 96 | 11.40 | 6.32 | 18.78 |
| 72 | 78.37 | 64.06 | 91.65 | 97 | 9.10 | 4.55 | 15.95 |
| 73 | 48.26 | 31.67 | 65.66 | 98 | 36.92 | 13.51 | 84.10 |
| 74 | 28.20 | 18.67 | 40.33 | 99 | 43.91 | 108.41 | 15.28 |
| 75 | 21.67 | 13.76 | 31.75 |  |  |  |  |
